# Simultaneous monitoring of SARS-CoV-2 and bacterial profiles from the air of hospital environments with COVID-19-affected patients

**DOI:** 10.1007/s10453-022-09754-7

**Published:** 2022-09-08

**Authors:** Maria Rita Perrone, Salvatore Romano, Giuseppe De Maria, Paolo Tundo, Anna Rita Bruno, Luigi Tagliaferro, Michele Maffia, Mattia Fragola

**Affiliations:** 1 Department of Mathematics and Physics, University of Salento, 73100 Lecce, Italy; 2 Presidio Ospedaliero Santa Caterina Novella, Azienda Sanitaria Locale Lecce, 73013 Galatina, Lecce, Italy; 3 Department of Biological and Environmental Sciences and Technologies, University of Salento, 73100 Lecce, Italy

**Keywords:** COVID-19, Hospital wards, Environmental microbiology, Air samplings, Bacterial profiles, 16S rRNA gene metabarcoding

## Abstract

The SARS-CoV-2 presence and the bacterial community profile in air samples collected at the Intensive Care Unit (ICU) of the Operational Unit of Infectious Diseases of Santa Caterina Novella Hospital in Galatina (Lecce, Italy) have been evaluated in this study. Air samplings were performed in different rooms of the ICU ward with and without COVID-19 patients. No sample was found positive to SARS-CoV-2, according to Allplex 2019-nCoV Assay. The airborne bacterial community profiles determined by the 16S rRNA gene metabarcoding approach up to the species level were characterized by richness and biodiversity indices, Spearman correlation coefficients, and Principal Coordinate Analysis. Pathogenic and non-pathogenic bacterial species, also detected in outdoor air samples, were found in all collected indoor samples. *Staphylococcus pettenkoferi, Corynebacterium tuberculostearicum*, and others *coagulase-negative staphylococci*, detected at high relative abundances in all the patients’ rooms, were the most abundant pathogenic species. The highest mean relative abundance of *S. pettenkoferi* and *C. tuberculostearicum* suggested that they were likely the main pathogens of COVID-19 patients at the ICU ward of this study. The identification of nosocomial pathogens representing potential patients’ risks in ICU COVID-19 rooms and the still controversial airborne transmission of the SARS-CoV-2 are the main contributions of this study.

**Supplementary Information:**

The online version contains supplementary material available at 10.1007/s10453-022-09754-7.

## Introduction

Bioaerosols, comprising living and dead microorganisms such as virus, bacteria, and fungi originated from humans, animals, plants, soil, food, or water, are a ubiquitous component of atmospheric air (Fröhlich-Nowoisky et al., 2016) and, including their patient-derived component, affect the hospital air quality, which is recognized as an important factor in most healthcare-associated infections (HCAIs). Comorbid infections caused by pathogenic and opportunistic microorganisms representing the environment microbioma have been detected in hospital wards (Pochtovyi et al., 2021), and several studies have been undertaken to characterize both viral and bacterial pathogens causing hospital infections (e.g. Yagoub & El Agbash, 2010). Kowalski and Bahnfleth (1998) provided a list of the primarily nosocomial respiratory bacterial pathogens. Nosocomial infections or HCAIs represent a challenging health problem around the world (Chezganova et al., 2021), since they affect 3–15% of patients within inpatient health-facilities according to recent estimates (Zingg et al., 2019) and may reach 30% of the patients in intensive care units (Magill et al., 2018). HCAIs increase deaths (morbidity and mortality) and antimicrobial resistance, prolong the duration of hospital stays and, consequently, raise healthcare costs (Ribeiro et al., 2019). Recently, it has also been shown that the SARS-CoV-2 (Severe Acute Respiratory Syndrome-CoronaVirus-2) infection is often accompanied by the occurrence of various comorbid infections, which worsen the disease course and increase duration of the patient’s stay in the hospital, as well as the probability of a fatal outcome (Bardi et al., 2021). SARS-CoV-2 was first diagnosed in Wuhan, China, in December 2019 (Forrester et al., 2020) and it is the causative agent of the 2019 coronavirus disease (COVID-19), which has spread and keeps spreading across many countries around the world. The World Health Organization (WHO) declared the SARS-CoV-2 infection outbreak as a pandemic on March 11, 2020. Several studies have been carried out to investigate the incidence of air bacterial and fungal coinfections in hospitalized patients affected by the COVID-19 disease (Hemati et al., 2021; Hughes et al., 2020; Marotz et al, 2021). The application of molecular biological techniques has represented a major step forward in the study of the airborne biota distribution in the environment (Yoo et al., 2017). More specifically, the 16S rRNA next-generation-sequencing (NGS) and the real-time reverse transcription polymerase chain reactions (RT-PCR) are commonly used to assess a broader diversity of bacteria, which are among the most frequent microorganisms in co-infections (Hemati et al., 2021; Pochtovyi et al., 2021; Rampelotto et al., 2019; Ribeiro et al., 2019). Pochtovyi et al. (2021) used the 16S rRNA Gene Amplicon Sequencing to investigate the contamination of hospital surfaces with bacterial pathogens in the First Moscow Infectious Diseases Hospital (Russia), one of the capital’s medical institutions designated for the treatment of patients with COVID-19. They found that the most contaminated surfaces were the floor and door handles. The most widespread microorganisms were the *coagulase-negative staphylococci* (*CoNS*), which were found in all samples. *Klebsiella pneumoniae* was identified in 100% of Intensive Care Unit (ICU) samples and in 64% of Respiratory Infectious Department (RID) samples. Thus, these two species were the main contaminants under the studied conditions. *Achromobacter spp.* (23%), *Staphylococcus aureus* (15%), and *Pseudomonas aeruginosa* (8%) were detected in the ICU. For RID, the distribution was slightly different, with *Pseudomonas aeruginosa* identified in 27% of the samples and *Achromobacter spp*. identified in 14% of samples, while no *Staphylococcus aureus* was detected. The study by Habibi et al. (2021) reported concentrations of SARS-CoV-2, other respiratory viruses, and pathogenic bacteria in the indoor air of the three major hospitals (Sheikh Jaber, Mubarak Al-Kabeer, and Al-Amiri) in Kuwait, dealing with COVID-19 patients. They used the RNA-based culture-independent approach to quantify viable bacterial cells that have direct health implications in indoor hospital air and pathogenic bacteria such as *Mycoplasma pneumoniae*, *Streptococcus pneumoniae*, and *Haemophilus influenzae* were found in the hospital aerosol samples.

In this study, indoor air samples have mainly been collected at the ICU of the Operational Unit (OU) of Infectious Diseases (ID) of Santa Caterina Novella-Hospital in Galatina (Lecce, Italy), designated for the treatment of patients with COVID-19 from January to June 2020. The detection/characterization of bacterial profiles in air samples collected in different areas of the ICU with hospitalized patients affected by the COVID-19 disease and the detection of the SARS-CoV-2 virus in air samples represent the main goals of the study. More specifically, the study aims to contribute to both the identification of nosocomial bacterial pathogens representing potential patient-level risk factors in ICU hospitalization rooms with and without COVID-19 patients and the still controversial airborne transmission of the SARS-CoV-2 (Ghaffari et al., 2021). The reported transmission modes of COVID-19 have mainly included the inhalation of liquid droplets produced by infected persons through direct or indirect person-to-person contacts. Nevertheless, the small liquid droplets can remain in the air and attach to aerosol particles, which may be spread by air movement to distances larger than the safe physical one (about 1.5 m), representing an additional route for the COVID-19 transmission (Liu et al., 2020; López et al., 2021; Setti et al., 2020).

Air samples were collected for this study from 30 April to 4 June 2020, since the hospitalization of COVID-19 patients in the ICU ward of the ID OU ended on 22 June 2020. One indoor sample in a hospitalization room of the Psychiatry Department and two outdoor samples on the roof of the ID OU were also collected to compare indoor and outdoor outcomes. The 16S rRNA gene partial sequencing was used to detect and classify the airborne pathogenic bacterial profiles, while a commercial kit (Allplex 2019-nCoVAssay) was used for the SARS-CoV-2 detection in air samples. Note that the contamination of hospital surfaces with bacterial pathogens and/or SARS-CoV-2 has already been investigated in most of previous studies and does not represent a purpose of this work.

## Materials and methods

### Air sample collection

The ACD-200 Bobcat (InnovaPrep LLC, Drexel, MO, USA; https://www.innovaprep.com, accessed on 24 February 2022) dry-filter air sampler was used to collect aerosol and biaoaerosol particles (Cox et al., 2020). It is a light-weight and portable device with a unique rapid filter elution kit, ideally suited for the collection of micron- and submicron-sized total suspended particles (TSPs), including viruses, bacteria, pollen, moulds, and fungal spores, as well as non-biological particles. More specifically, the ACD-200 Bobcat uses a dry 52-mm-diameter electret filter as collection medium (Shu et al., 2017); each electret filter is equipped with a filter holder contained in a sterilized box before being inserted into the ACD-200 Bobcat air sampler using sterile latex gloves. Electret filters are made up of polymer fibres that carry a permanent positive and negative charge, which increases the collection efficiency allowing higher sampling rates for extended periods also using a battery power. The filter-holder system at the end of the collection time interval is removed from the collector using sterile latex gloves, snapped onto the sample cup, and fitted with the elution adapter. To extract the captured particles from the filter, the user presses the canister containing the Wet Foam Elution™ patented by InnovaPrep LLC, then the elution foam is released from the canister evenly through the filter and passes through the interstitial spaces of it to efficiently extract any captured particles. Sample elution takes approximately 5 s and produces 6 to 7 millilitres of liquid sample. The foam immediately collapses back to a liquid phase in the sample cup, making it available for sample processing and analysis.

### Sampling site and main characteristics of the collected air samples

Air samples have mainly been collected at the ID OU of Santa Caterina Novella-Hospital in Galatina (Lecce, Italy), which is a small town (40.1°N; 18.1°E) of the Salento peninsula in South-eastern Italy. The schematic view of the Intensive Care Unit of the ID OU, where COVID-19 patients were located, is shown in Figure S1. It consists of the Medicine-Store Room (MSR), the doctor’s office, five Rooms for Patients (RPs) with conventional air conditioning, and three negative-pressure High-Isolation-Rooms (HIRs) with 25 air changes/hour leading to about 800 m^3^/h of outdoor air intake without using filter system, while the exhaust air is filtered through HEPA-H13 filters. All ICU rooms are equipped with a BaThroom (BT). The visitors’ access to the ICU was completely restricted. The number of COVID-19 patients in the ICU ward varied from 9 to 5 during the sampling time interval of this study, which lasted from 30 April to 4 June 2020 because of technical and bureaucratic restrictions. Twelve samples have been collected in different rooms/areas of the ICU ward by the ACD-200 Bobcat air sampler, as listed in Table 1. Samples have been denoted in order to make easier for the reader to track the sampling location. More specifically, the letters HIRP and RP have been used to identify samplings performed, respectively, in a High Isolation Room and a conventional Room for Patients with patients. Then, a capital letter (A, B, C) in subscript has been used to identify the presence of one or two patients. The capital letter P has been left out in case of measurements performed in ICU rooms not occupied by patients. Seven samples were collected in rooms with COVID-19 patients, while five samples were collected in rooms without any patient. Therefore, sample-HIRP_A_ was collected in a HIR occupied by Patient A. Sample RP_A_ was collected in the RP where patient A was moved from the HIR. Samples RP1_B_ and RP2_B_ were collected in the RP occupied by patient B, whereas sample BTP_B_ was collected in the BaThroom of the RP occupied by patient B. Samples RP1_B+C_ and RP2_B+C_ were collected in the RP occupied by patients B and C. Samples HIR1 and HIR2 were collected in a HIR without any patient, while samples R1 and R2 were collected in RPs without any patient. Sample-MSR was collected in the Medicine-Store Room, only accessed by medical staff and health care operators. Two 52 mm-diameter dry electret filters located in the corridor at about 1 m from the floor (EF1) and in a patient room about 1 m away from the patients’ bed (EF2) have allowed collecting aerosol and bioaerosol particles from gravimetric deposition over 14 days. Two air samples were also collected on the Roof of the ID OU Building in May (RB1) and July (RB2) to characterize the bacterial profile of outdoor air samples and investigate their relationships with indoor samples. One air sample (PSD) was also collected on 15 July 2020 in a hospitalization room of the PSychiatry Department, which is on a different floor of the ID OU building, for the comparison with the ICU samples. Table 1 provides the list of the 17 collected samples with the corresponding sampling date, bacterial read concentration, and bacterial taxonomic rank. Samples are ordered in accordance with the monitoring room/area characteristics and the sampling type to better highlight the sampling feature impact on the bacterial taxonomic rank of the collected samples.

**Table 1:** Read concentrations (expressed as reads/m^3^) at phylum level and number (n°) of identified bacterial phyla, classes, orders, families, genera, and species in the 17 collected samples. The sampling date of each sample is also reported

Sample	Date (dd/mm/yy)	Reads/m^3^ (at phylum-level)	n° phyla	n° classes	n° orders	n° families	n° genera	n° species
HIRP_A_	30/04/20	4364	36	67	101	260	880	1471
RP_A_	01/05/20	5140	38	74	113	269	1001	2016
RP1_B_	05/05/20	6791	35	66	98	257	953	1804
RP2_B_	07/05/20	3958	37	74	111	281	1092	2311
BTP_B_	06/05/20	3229	32	71	104	263	914	1875
RP1_B+C_	17/05/20	1162	41	83	121	306	1333	3182
RP2_B+C_	21/05/20	869	41	83	125	317	1461	3737
EF1	07/05/20	–	37	78	118	291	1031	1965
EF2	07/05/20	–	39	77	114	282	1095	2202
HIR1	01/05/20	3515	30	65	99	248	781	1133
HIR2	15/05/20	88	39	83	117	291	1343	3301
R1	02/05/20	5645	41	84	123	306	1399	3397
R2	04/06/20	746	41	82	115	301	1256	2898
MSR	03/05/20	16	12	29	48	98	143	126
RB1	08/05/20	325	37	77	102	265	919	1650
RB2	16/07/20	467	40	79	117	301	1350	3291
PSD	11/07/20	910	42	80	122	310	1449	3607

### SARS-CoV-2 detection methodology

The Allplex 2019-nCoV Assay (Seegene Inc., South Korea; https://www.seegene.com/assays/allplex_2019_ncov_assay, accessed on 24 February 2022) was used for the qualitative detection of nucleic acid from Severe Acute Respiratory Syndrome-related CoronaVirus 2 (SARS-CoV-2) in 2–3 millilitres of each collected liquid sample. The Allplex 2019-nCoV Assay is an in vitro diagnostic real-time reverse transcriptase polymerase chain reaction (RT-PCR) test, commonly used to test human nasopharyngeal swab, oropharyngeal swab, anterior nasal swab, mid-turbinate, and sputum specimens from individuals with signs and symptoms of infection and who are suspected of COVID-19 by their health care provider. A positive and a negative control were used in each sample amplification session to validate the session itself. The Allplex 2019-nCoV Assay has an analytical sensitivity of 50 copies per reaction, i.e. about 3 viral copies per microliter of sample. The test was performed few hours after each sample collection; then the remaining liquid sample was stored at about − 30 °C for additional analyses.

### DNA extraction and 16S rRNA gene high-throughput sequencing

The seventeen liquid samples were processed using the DNeasy PowerWater kit (Qiagen, Milan, Italy), and the genomic DNA was extracted following the manufacturer’s recommendations, then stored at − 30 °C until further analysis.

High-throughput sequencing experiments and primary bioinformatics analyses were performed by Genomix4life S.R.L. (Baronissi, Salerno, Italy), as described in Romano et al. (2019). Briefly, DNA quantity and quality were assessed using both NanoDropOne spectrophotometer (Thermo Scientific, Waltham, MA) and Qubit Fluorometer 4.0 (Invitrogen Co., Carlsbad, CA). The hypervariable V3 and V4 regions of the 16S rRNA gene were then subjected to PCR amplification with primers: Forward: 5’-CCTACGGGNGGCWGCAG-3’ and Reverse: 5’-GACTACHVGGGTATCTAATCC-3’ (Klindworth et al., 2013). Each PCR reaction was assembled in agreement with 16S Metagenomic Sequencing Library Preparation (Illumina, San Diego, CA). Libraries were quantified by Qubit fluorometer (Invitrogen Co., Carlsbad, CA) and pooled to an equimolar amount of each index-tagged sample up to a 4 nM concentration, including the Phix Control Library. Pooled samples underwent cluster generation and were sequenced on MiSeq platform (Illumina, San Diego, CA) in a 2 × 250 paired-end format. The generated raw sequence files (fast files) were finally subjected to quality control with FASTQ. A negative control, consisting of all the reagents used over each sample processing but without DNA template, was included in the workflow to ensure no contamination. More specifically, the negative control performed in the various steps of the amplicon generation and subsequent generation of the indexed library did not produce any analysable library. The sequencing of the negative control library also did not produce analysable reads.

The taxonomic classification of amplicon 16S rRNA-gene reads was performed by means of the 16S Metagenomics app (Illumina, Version 1.1.0), a high-performance implemented algorithm of the Ribosomal Database Project (RDP) classifier (Wang et al., 2007). The taxonomic database used by the algorithm is RefSeq RDP 16S v3 May 2018 DADA2 32 bp (Alishum, 2019). Any reads not matching a reference sequence were considered as unclassified and were also reported in the bacterial community analysis.

### Statistical analysis methodologies

The number of OTUs (operational taxonomic units), the Shannon and Simpson indices (*H* and *D*, respectively), and the Principal Coordinate Analysis (PCoA) represent the most common tools to investigate richness, biodiversity, and similarity, respectively, among samples of a specific environmental dataset at a given taxonomic level. We used these tools, in addition to the Spearman’s rank-order correlation coefficients, to characterize the structure of the airborne bacterial community at the species level for all samples.

The relationships between the most abundant bacterial species identified in our PM samples were investigated using the non-parametric Spearman’s rank-order correlation coefficients, estimated by means of the PAST (Paleontological Statistics) software package (Hammer et al., 2001). The not-normal distribution of the investigated parameters was verified by the Kolmogorov–Smirnov test (using the MATLAB *kstest* function), because Spearman coefficients do not rest upon the assumption of data normality.

The biodiversity of our PM samples at the species level was then evaluated using the Shannon *H* (Shannon, 1948) and Simpson *D* (Simpson, 1949) indices, commonly used to quantify and describe the community (alpha) diversity (e.g. Escobar-Zepeda et al., 2015; Kim et al., 2017). In detail, the two biodiversity indices investigated in this study were calculated as follows:

1 $$H = - \Sigma_{i} p_{i} \ln p_{i}$$

2 $$D = \Sigma_{i} \left( {p_{i} } \right)^{2}$$

where *p*_*i*_ is equal to *n*_*i*_/*N* for a well-sampled community, with *n*_*i*_ representing the number of individuals in the species *i* and *N* the corresponding total number in all the community (Krebs, 2014). The value of *H* is strictly proportional to the diversity of species in a particular community: the higher the value of *H*, the higher the diversity of species. On the contrary, *H* = 0 indicates a community that only has one species. *D* is a measure of diversity that considers both the number of species present in a specific community and the corresponding relative abundance of each species. Therefore, if *D* increases, both species richness and evenness increase.

Finally, the Principal Coordinate Analysis (PCoA) technique was also used to study the relationships among the bacterial communities identified in the collected samples at the species level. The PCoA is currently one of the most common ordination methods for exploratory analyses since it allows the graphical representation of the similarity among values of more variables (Ramette, 2007). More specifically, PCoA components represent a complex function of original variables used as input data, based on a given distance matrix (Hervé et al., 2018). Note that in our study we used the Bray–Curtis distance matrix obtained from the relative abundances of the most abundant species identified in our PM samples as input parameter for the PCoA analysis. The PCoA performance can be generally evaluated using the percentage of the total variance explained by the first two axes related to the corresponding components. We applied the PCoA ordination method to the selected datasets by means of the PAST software package (Hammer et al., 2001).

## Results and discussion

### SARS-CoV-2 detection in air samples

None of the air samples collected in the ICU ward of this study was found positive to SARS-CoV-2, according to the Allplex 2019-nCoV Assay which has an analytical sensitivity of 50 copies per reaction (3 viral copies per microliter of sample). Our results are consistent with those from previous studies, which detected SARS-CoV-2 at very low rates, likely for weak ability of the virus to be transmitted by aerosol particles (Ghaffari et al., 2021; Hemati et al., 2021; Liu et al., 2020; López et al., 2021; Zhang et al., 2021). The first study on the SARS-CoV-2 detection in air samples was performed by Liu et al. (2020) using a droplet digital Polymerase Chain Reaction (ddPCR) method, which was carried out according to the manufacturer’s instructions for the QX200 Droplet Digital PCR System (Bio-Rad). The lower limit of detection (LLoD) of the optimized ddPCR was of 2.18 copies per reaction and 0.42 copies per reaction (20 µl) for ORF1ab and N primers/probe sets, respectively. They collected 35 aerosol samples in different patient rooms and/or contaminated areas of the Renmin and Wuchang Fangcan Hospital in Wuhan (China). Undetectable or low airborne SARS-CoV-2 concentrations were found in the patient areas of both hospitals. The air sample collected in a patient mobile toilet room of the Fangcan hospital tested significantly positive. López et al. (2021) have investigated the detection of SARS-CoV-2 in air samples collected in two hospitals in Hermosillo (Mexico) by trapping aerosol particles on 0.22 μm-pore filters, which were macerated using nuclease-free disposable plastic pistils to obtain total RNA. For the diagnosis of SARS-CoV-2, total RNA was processed through a real-time RT-PCR technique with commercial reagents (WoV19 Kit), according to the protocol described by Corman et al. (2020). The RNA was extracted applying a commercial kit (RNAspin miniRNA Isolation kit, G.E. 25–0500-71) following the manufacturer’s instructions. All assays were highly sensitive, with best results obtained for the E gene and RdRp gene assays (5.2 and 3.8 copies per reaction (25 µl)) at 95% detection probability, respectively (Corman et al., 2020). Their results confirmed the possibility of finding this virus in aerosol particles. Ghaffari et al. (2021) investigated the presence of SARS-CoV-2 in air samples collected at the Shahid Mohammadi Hospital Complex of Bandar Abbas (Iran). The presence of SARS-CoV-2 genome was assessed using a commercially available SARS-CoV-2 Test Kit (Pishtaz-Iran), according to the manufacturer’s instructions using One Step plus Real-Time PCR system tool (Applied Biosystems, USA). The SARS-CoV-2 Test Kit is a molecular in vitro diagnostic test that uses Taqman probe-based technology for the qualitative detection of SARS-CoV-2. The N and RdRp genes were the target for the detection of the virus, and RNase P was also used as an internal control. This also served as the extraction control to ensure that samples resulting as negative contained extracted nucleic acid for testing. The positive control must be positive at a Ct value of 30.96 ± 2.00. The LLoD of the used method was of 200 copies per mL of sample (https://labtechniche.com/wp-content/uploads/PCR-Covid.pdf, accessed on 21 March 2022). Only 2 out of the 16 collected air samples were found positive, of which one was taken near COVID-19 patient’s bed. Both samples were taken from the places which had higher concentrations of PM. Zhang et al. (2021) collected 12 bioaerosol samples and 7 swab samples from the environments hosting COVID-19 patients in ICU hospital wards. DNA was extracted and purified from 200 μl aerosol samples or swabs according to the instruction of DNA Mini kit DP316 (Tiangen). RNA was extracted by QIAamp Viral RNA Mini Kit 52,906 (Qiagen), following a reverse transcription using SMART MMLV Reverse Transcriptase kit 639,524 (Takara). The concentration and quality of DNA and cDNA were checked using Qubit (Thermo Fisher). They found a very low detection rate of SARS-CoV-2 by mNGS in the aerosol and swab samples. Indeed, only one aerosol sample collected at a distance < 0.5 m from the patient was detected positive for COVID-19. Hemati et al. (2021) collected 45 air samples in the ICU ward of the Hajar Hospital in Shahrekord (Iran) by using a standard midget impinger containing 20-mL viral transport medium and found that 13.33% of the samples were positive to SARS-CoV-2 using the real-time PCR. RNA was extracted using the RNJia Virus Kit (Roje-Technologies, Yazd, Iran) according to the manufacturer’s protocol. Real-time PCR was conducted for SARS-CoV-2 RNA-dependent RNA polymerase (RdRp) on the FAM Channel and the nucleocapsid protein (NP) on the HEX Channel using the Novel Coronavirus (2019-nCoV) Nucleic Acid Diagnostic Kit (Pishtazteb Zaman Diagnostics, Tehran, Iran). The LLoD of the used diagnostic kit was established using both assays that presented equivalent results of 200 copies per mL of sample. However, the airborne transmission of SARS-CoV-2 is still a debated issue and more studies are required to unravel all virus dissemination routes in the present pandemic. Presumably, rather high concentrations of aerosol particles are required and/or the positivity of some molecular tests are insufficient to draw any conclusion on the SARS-CoV-2 airborne transmission, according to Barbieri et al. (2021).

### Bacterial taxonomic ranks in the collected air samples

Table 1 shows the read concentration (expressed as reads/m^3^) due to bacterial phyla, and the number (n°) of identified phyla, classes, orders, families, genera, and species in the 17 collected air samples, in addition to their sampling date, providing a preliminary overview of the samples’ bacterial taxonomic rank characterization. All listed parameters vary from sample-to-sample. A special attention is to be paid to sample MSR, where all taxonomic rank parameters reached the smallest value in number (Table 1), suggesting that the Medicine-Store Room was likely the least contaminated and, consequently, MSR has been assumed as control sample. The number of identified species, which was equal to 126 in sample MSR, varied within the 1,133–3,737 range in all the other samples.

### Overview of the bacterial communities at the phylum level

**Fig. 1:**
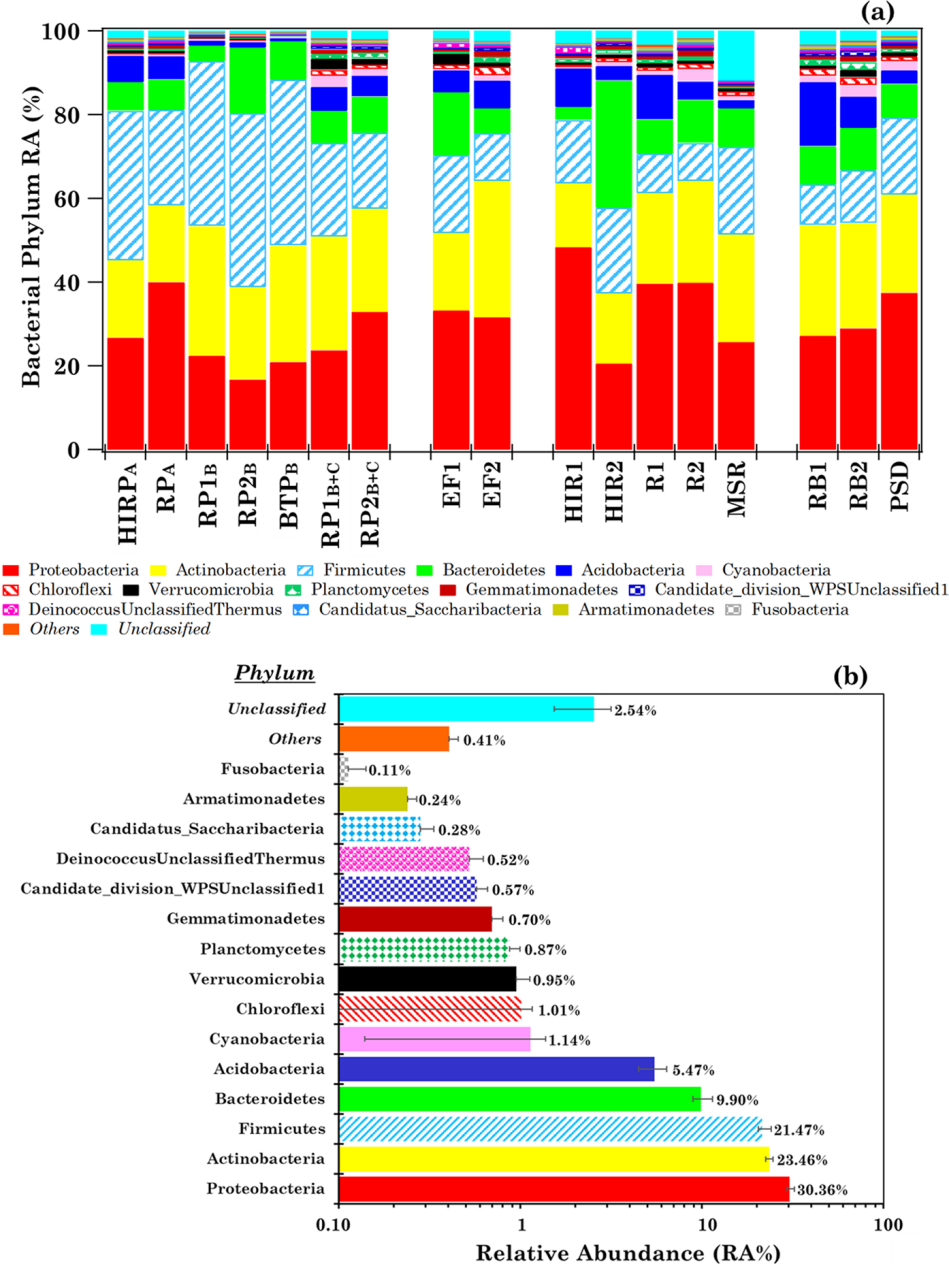
**a** Within-sample relative abundance percentage (RA%) of the 15 most abundant bacterial phyla detected in the 17 analysed samples with mean RA% > 0.11% and **b** their mean percentage contributions. The bacterial phyla with a mean RA% ≤ 0.11% range and the unclassified ones (denoted as “*Others*” and “*Unclassified*”, respectively) are also represented in each plot. Error bars in (**b**) represent the standard error of the mean

The number of detected phyla varied from 30 to 42 in the collected samples, except in sample MSR where only 12 phyla were detected (Table 1). The heat-map of the within-sample phylum relative abundances (RAs%) is shown in Table S1, where samples are ordered in accordance with the monitoring room/area characteristics and the sampling type. Figure 1a shows the within-sample RA% of the 15 most abundant bacterial phyla (mean RA% > 0.11%) in the collected samples. The mean percentage contribution of each phylum (on a logarithmic scale) is shown in Fig. 1b. The bacterial phyla with a mean RA ≤ 0.10% and the unclassified ones (denoted as *Others* and *Unclassified*, respectively) are also represented in each plot of Fig. 1. *Others* and *Unclassified* mean RAs% are equal to 0.41% and 2.54%, respectively. The 15 most abundant phyla were detected in all samples, apart from Candidatus-Saccharibacteria, Armatimonadetes, and Fusobacteria, which have not been detected in sample MSR. Proteobacteria, Actinobacteria, Firmicutes, and Bacteroidetes were the prevailing phyla in all samples, reaching a mean RA% of 30, 23, 21, and 10%, respectively, across the collected samples. These results are consistent with previous studies performed in hospital ICU wards (e.g. Ribeiro et al., 2019; Zhang et al., 2021).

Proteobacteria, Actinobacteria, Firmicutes, and Bacteroidetes were also the prevailing phyla in the two outdoor samples of this study (RB1 and RB2), in good accordance with the results by Romano et al. (2019). Figure 1 results are also consistent with previous outdoor studies performed not only in the Mediterranean (Mazar et al., 2016), but also in different sites (e.g. Gao et al., 2017; Mayol et al., 2017). In fact, the above-listed prevailing phyla could be found at high relative abundances in aquatic and soil environments and, consequently, in both indoor and outdoor monitoring sites.

The bacterial community profile at lower hierarchy is expected to be significantly dependent on the sampling site features, as it will be shown in the following.

### Genus-level bacterial communities in the collected samples

**Fig. 2:**
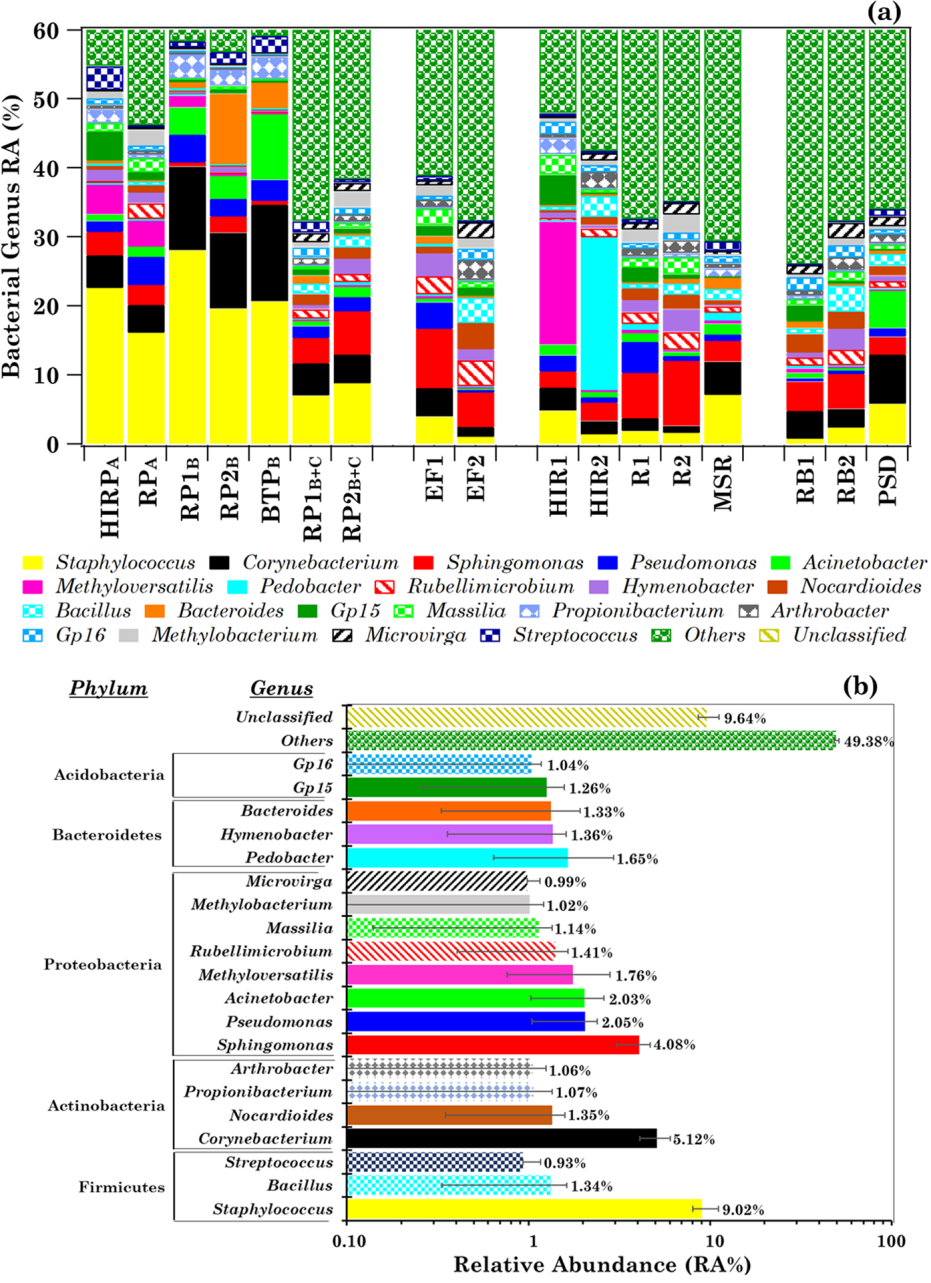
**a** Within-sample relative abundance percentage (plotted up to RA% = 60%) of the 20 most abundant bacterial genera detected in the 17 analysed samples with mean RA% > 0.93% and **b** their mean percentage contributions. The bacterial genera with a mean RA% ≤ 0.93% and the unclassified ones (denoted as “*Others*” and “*Unclassified*”, respectively) are also represented in (**b**), where error bars represent the standard error of the mean. The corresponding phylum related to each genus is also reported in (**b**)

The number of detected genera varied from 781 to 1,461 in the 17 collected samples, except in sample MSR where only 143 genera were detected (Table 1). The heat-map of the first 100 most abundant genera (mean RA% > 0.14%) is shown in Table S2. Figure S2 shows the within-sample RA% of the 20 most abundant genera (mean RA% > 0.93%). Figure 2a displays the within-sample RAs% up to 60% to better show the sample-to-sample changes of the 20 bacterial genus within-sample RAs%. Their mean percentage contribution is shown in Fig. 2b. The RAs% of all genera with mean RAs% ≤ 0.93% and the unclassified ones, denoted as *Others* (mean RA% = 49.38%) and *Unclassified* (mean RA% = 9.64%), respectively, are also shown in Fig. 2a, b. *Staphylococcus* was the most abundant genus in ICU rooms with patients and in the Medicine Store Room (sample MSR). It was also the second most abundant genus in sample PSD, which was collected in a patient room of the Psychiatry Department. The genus *Staphylococcus* currently includes more than 45 species, mostly commensals of the skin and mucous surfaces of humans and other mammals. It is widespread in the environment and was detected in all indoor and outdoor samples of this study at RAs% on average higher than few percent. It is responsible for infections especially in patients with prolonged hospitalization, comorbidities, and in patients undergoing prolonged antibiotic therapy (Chezganova et al., 2021; Ribeiro et al., 2019). *Staphylococcus*, *Corynebacterium*, *Streptococcus*, and *Acinetobacter* are considered as the dominant genera in hospital wards (Lax et al., 2017; Zhang et al., 2021), in reasonable accordance with the results of this study. *Corynebacterium*, the second most abundant genus in the collected samples of this study, has also been detected in all samples and on average with RAs% higher than few percent in patient rooms. It is also a skin colonizer sometimes isolated in patients with prolonged hospitalization (Sirivongrangson et al., 2020; Zhang et al., 2021). The third predominant genus was *Sphingomonas* (Fig. 2b), which is also responsible for hospital-associated infections from environmental exposure (Sirivongrangson et al., 2020). The genus within-sample RAs% varied differently among patient rooms and more generally among all analysed samples (Fig. 2a), likely because several local and environmental factors contributed to the sample bacterial profiles. Most of the genera listed in Fig. 2a were also detected in one or more studies referring to the characterization of the genus community in hospital environments (Li et al., 2021; Ribeiro et al., 2019; Zhang et al., 2021).

The quite high RA% that *Methyloversatilis* (17.88%) and *Pedobacter* (22.39%) genera reached in samples HIR1 and HIR2 (Fig. 2a), respectively, deserves special attention, also because both can be considered as non-pathogenic genera (e.g. Kalyuzhnaya et al., 2006; Margesin & Shivaji, 2015). *Methyloversatilis* was found in a variety of natural and engineered ecosystems. In more detail, Kalyuzhnaya et al. (2006) were able to isolate *Methyloversatilis* species from soils contaminated with chemical industrial wastes, while it was detected in sparkling natural mineral water by Brumfield et al. (2020) and in hospital sinks by D'Arcy (2014). *Methyloversatilis* was detected in all samples of this study except in the PSD sample. The outdoor environment likely contributed to the rather high *Methyloversatilis* within-sample RA% in HIR1. Indeed, HIR rooms, as mentioned, were subjected to 25 outdoor-air exchanges/hour, without any filtering system, leading to about 800 m^3^/h of outdoor air intake. *Pedobacter*, whose within-sample RA% was highest in sample HIR2, has commonly been identified in soil, activated sludge, glacier cryoconite, and freshwater (e.g. Dahal & Kim, 2016; Margesin & Shivaji, 2015; Margesin et al., 2003). Recently, Samaké et al. (2020) have investigated the PM10-associated microbial communities at three sampling sites in France, under different climates, during summer in 2018 and they found that *Pedobacter* was among the most abundant genera. Again, we believe that the about 800 m^3^/h of outdoor air intake during the HIR2 sampling have likely contributed to *Pedobacter* diffusion in the sampling room and, therefore, to its rather high within-sample RA% in HIR2.

The high particulate matter (PM) concentration in the outdoor air monitored during the HIR2 sampling may support our speculation. In fact, according to the data provided by the Regional Air quality Agency (ARPA) of the Puglia Region for the study site (Galatina, Lecce), the PM10 mass concentration, which was equal to 18 μg/m^3^ on 14 May, increased up to 47 μg/m^3^ on 15 May 2020 (http://old.arpa.puglia.it/web/guest/qariainq, accessed on 24 February 2022), which was the HIR2 sampling day. The intense desert dust outbreak, which affected South-eastern Italy during the HIR2 sampling, according to the BSC-DREAM M8b v2.0 model developed at the Barcelona Super Computing Centre (https://ess.bsc.es/bsc-dust-daily-forecast, accessed on 24 February 2022), likely contributed to this result. Figure S3, which displays by a colour map the dust load (expressed as g/m^2^) over Europe and Northern Africa provided by the model on 15 May 2020 at 12:00 UTC, shows that the dust load over South-eastern Italy was within the 1.0–1.5 g/m^2^ range, significantly greater than in other Southern Italy regions.

### Overview of the bacterial community at the species level

**Fig. 3:**
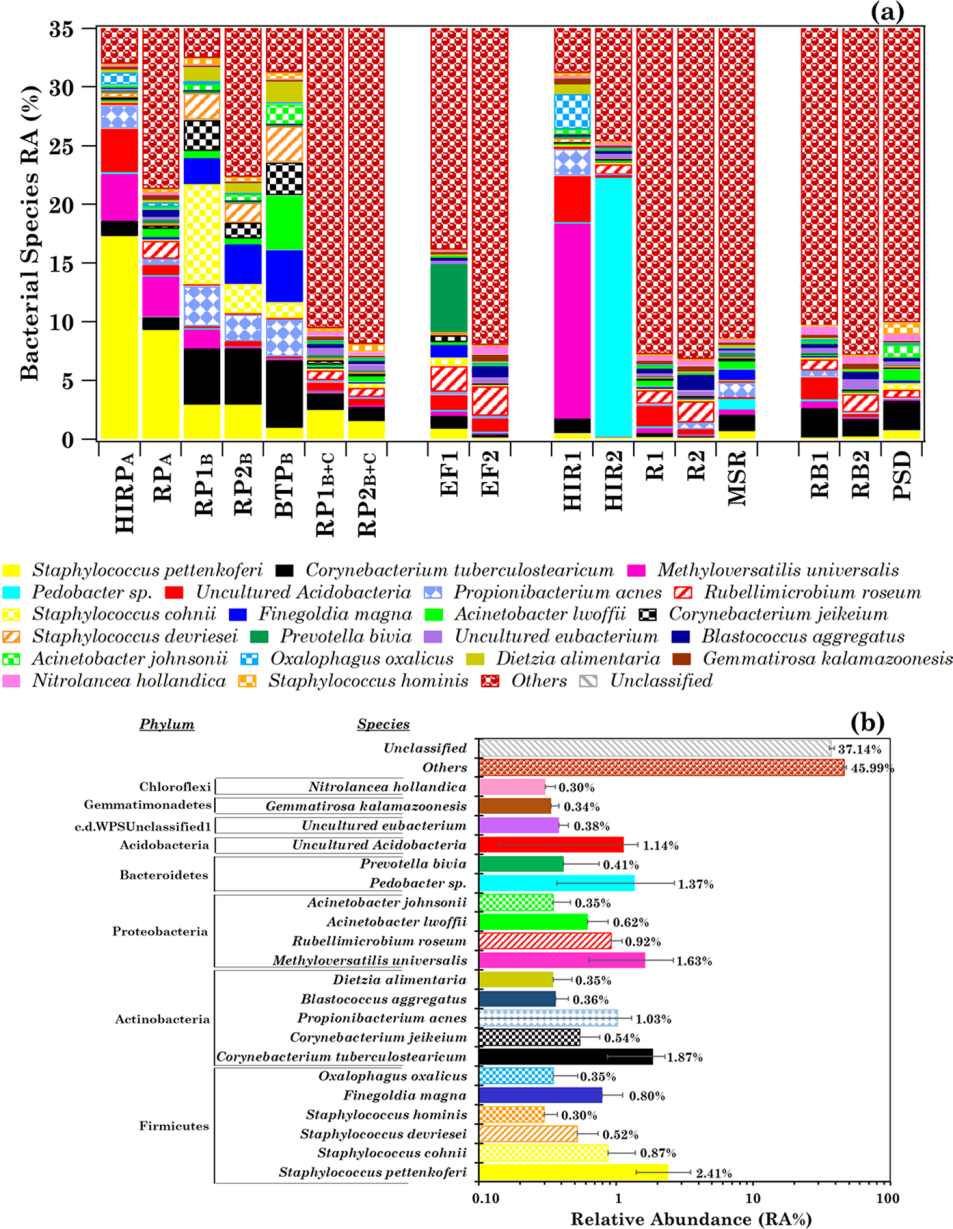
**a** Within-sample relative abundance percentage (RA%, plotted up to 35%) of the 21 most abundant bacterial species with mean RA% > 0.30% detected in the 17 analysed samples and **b** their mean percentage contributions. The bacterial species with a mean RA% ≤ 0.30% and the unclassified ones (denoted as “*Others*” and “*Unclassified*”, respectively) are also represented in the plot. The error bars in (**b**) represent the standard error of the mean. The corresponding phylum related to each species is also reported in (**b**)

The sample bacterial-species-profiles are analysed in more detail in this section, since the differences among samples increase at lower hierarchy. Table 1 shows that the number of detected species in each sample varied within the 1,133–3,737 range, except in sample MSR where only 126 species were detected. More specifically, 6,142 different species have been detected in the 17 analysed samples. Table S3 shows the sample heat-map of the 100 most abundant species (mean RA% > 0.10%), which have on average been detected in more than 52% of the samples. The within-sample RA% of the 21 most abundant species (mean RA% > 0.30%), which have on average been detected in more than 70% of the samples, is shown in Figure S4, where *Others* provides the within-sample RA% of the remaining species, which varied within 32.11–57.99% in the analysed samples. The within-sample RAs% of the *Unclassified* species, which varied within the 27.27–59.40% range, is also shown in Figure S4. Figure 3a shows the sample RAs% up to 35% to better highlight the within-sample RAs% of the 21 most abundant species. Their mean RA% is shown in Fig. 3b. *Staphylococcus pettenkoferi* (mean RA% = 2.4%) and *Corynebacterium tuberculostearicum* (mean RA% = 1.9%) were the two most abundant pathogenic species. *Staphylococcus pettenkoferi* is a *coagulase-negative staphylococcal* (*CoNS*) species isolated from human clinical specimens (Trülzsch et al., 2002, 2007). *CoNS* species are among the most commonly isolated bacterial species in clinical microbiology laboratories, undoubtedly mostly as skin contaminants, as mentioned in the previous section. They are of increasing importance as nosocomial pathogens, since they infect especially immunocompromised patients (Chezganova et al., 2021; Ribeiro et al., 2019; Zhang et al., 2021). In addition to *Staphylococcus pettenkoferi*, *Staphylococcus cohnii* (mean RA% = 0.87%), *Staphylococcus devriesei* (mean RA% = 0.52%), and *Staphylococcus hominis* (mean RA% = 0.30%) have also been detected among the 21 most abundant species. All detected *CoNS* species reached the highest RAs% in patients’ rooms (Table S3). More specifically, *Staphylococcus pettenkoferi* reached the highest within-sample RA% in the samples HIRP_A_ (17.31%) and RP_A_ (9.30%). Conversely, *S. cohnii*, S*. devriesei*, and *S. hominis* reached the highest within-sample RAs% in samples RP1_B_, RP2_B_, and BTP_B_, respectively. *Staphylococcus pettenkoferi* was among the main pathogens associated with COVID-19-affected patients at the Chinese PLA General Hospital, Beijing (China), according to Zhang et al. (2021). *S. cohnii*, the second most abundant among *CoNS* species in this study, is a widespread species in hospital environments (Szewczyk et al., 2004) and isolates of this bacterium were found next to *S. epidermidis*, *S. hominis*, and *S. haemolyticus*. *S. cohnii* strains gained the antibiotic resistance, which enables their long-lasting existence in the hospital wards, according to Szewczyk et al. (2004). Therefore, the real risk of the wide antibiotic resistance of *S. cohnii* is also due to its role as a carrier of the resistance genes to more pathogenic species. *S. pettenkoferi* has been detected in all indoor and outdoor samples of this study, while *S. cohnii*, *S. devriesei*, and *S. hominis* have been detected in most of the indoor samples and in the RB1 and RB2 outdoor samples, apart from *S. cohnii*, which was not found in RB1. Therefore, all the above-mentioned *CoNS* species were also spread in outdoor air, but hospital environments likely favoured their rapid proliferation and selection.

*Corynebacterium tuberculostearicum*, the second most abundant bacterial species, was also detected in all indoor and outdoor samples of this study. More specifically, it reached the highest within-sample RAs% in samples RP1_B_, RP2_B_, and BTP_B_ (Fig. 3), but within-sample RAs% similar to the ones of indoor samples were also detected in the outdoor RB1 and RB2 samples (Table S3). In fact, *C. tuberculostearicum* is a ubiquitous bacterium colonizing human skin, thus playing a role in skin diseases in a hospital environment (Altonsy et al., 2020). *Finegoldia magna* has also been detected at greater within-sample RAs% in RP1_B_, RP2_B_, and BTP_B_ samples. It contributes to the commensal flora of the skin, oral cavity, gastrointestinal tract, and the female urogenital tract (Frick et al., 2008). Note that *Acinetobacter lwoffii* reached in BTP_B_ a within-sample RA% (4.63%) more than four times greater than in all the other samples. This result could likely be supported by the work of Regalado et al. (2009), which reported a first case of a community-acquired *A. lwoffii* bacteremia associated with gastroenteritis. *A. lwoffi* is a potential opportunistic pathogen in patients with impaired immune systems and has been considered responsible for nosocomial infections like septicemia, meningitis, and pneumonia.

The *Corynebacterium jeikeium* species was detected in all samples of this study with a mean RA% = 0.54%. It is a multidrug-resistant gram-positive bacterium of the human skin flora and one of the most clinically important nondiphtherial corynebacteria in the acute care setting (Moore Pardo et al., 2020) and can cause infections, especially in immunocompromised patients with comorbidities. *Propionibacterium acnes* is a saprophyte of the skin, which generally has been implicated in acne inflammation and pathogenesis (Bourdeaut et al., 2002). Nowadays, *P. acnes*, also known as *Cutibacterium acnes* (Mayslich et al., 2021), is emerging as an important opportunistic pathogen and it is now the second most frequent pathogen, after *CoNS*, and rates of infection due to this bacterium have increased from 1.5 to 38% according to recent studies (Bayo et al., 2021; McDowell et al., 2021; Walti et al., 2013). *P. acnes* has been detected in all samples and at within-sample RAs% spanning the 1.97–3.41% range in the patient-room samples HIRP_A_, RP1_B_, RP2_B_, and BTP_B_, in addition to samples HIR1 (RA% = 2.30%) and MSR (RA% = 1.38%). Its within-sample RA% was ≤ 0.57% in all the other samples.

Aiyanyor et al. (2021) have recently reported the *Prevotella bivia* bacteremia developed in a patient with severe COVID-19 pneumonia following tocilizumab, and it is worth noting that a high within-sample RA% (5.78%) of *Prevotella bivia* was only detected in sample EF1, as Fig. 3a clearly shows. The EF1 sample collected aerosol and bioaerosol particles by gravimetric deposition over 14 days on a dry filter located in the corridor at about 1 m from the floor. *Methyloversatilis universalis* and *Pedobacter sp.* also reached quite high RAs% in HIR1 (16.60%) and HIR2 (22.60%), respectively (Fig. 3a). Moreover, *Rubellimicrobium roseum* reached the highest within-sample RAs% in the indoor samples EF1 (2.19%) and EF2 (2.52%) and in the outdoor sample RB2 (1.55%). It was one of the most ubiquitous soil and organic material-dwelling bacteria in outdoor PM, according to Kováts et al. (2019). In fact, they have analysed the microbial community of both inhalable and respirable fractions of the air resuspended dust collected in Budapest (Hungary). Their results can support the finding of this study, since EF1 and EF2 were collected from the gravimetric deposition of aerosol and bioaerosol particles over 14 days and the RB2 sample was collected during a dusty day. *Uncultured Acidobacteria* is a species of the Acidobacteria phylum whose members represent a significant fraction of soil microbial community, pervasively and copiously distributed across nearly all ecosystems. Consequently, the highest within-sample RAs% that *Uncultured Acidobacteria* reached in HIR1 (4.08%), HIRP_A_ (3.88%), and RB1 (2.06%) were likely associated with air resuspended dust.

In conclusion, pathogenic and non-pathogenic bacterial species also present in outdoor air have been detected in the analysed samples. The species within-sample RAs% varied significantly among samples or group of samples. The impact of the outdoor air quality on the within-sample RAs% of non-pathogenic species mainly in HIR has also been demonstrated. The comparison of the pathogenic bacterial species profiles of this study with the corresponding ones of other nosocomial sites with and/or without COVID-19 patients has shown the high variability of the prevailing pathogenic bacterial species among different study sites because of the environmental condition impact.

#### Relationships between the 21 most abundant bacterial species

The relationships among the 21 most abundant bacterial species detected in the 17 samples have been investigated by Spearman correlation coefficients (Table S4) to support and/or contribute to a better understanding of the previous Section results. Table 2 summarizes very strong and strong correlations between species associated with Spearman coefficients significant at a *p*-level < 0.05 (*) and < 0.01 (**), respectively. The 21 analysed species were positively and/or negatively correlated with one or more other species, except *Pedobacter sp.* and *Prevotella bivia*, since their respective highest within-sample RAs% were likely due to specific sampling conditions. *Prevotella bivia* was mainly detected in sample EF1, while *Pedobacter sp.* reached the highest within-sample RA% in sample HIR2, likely because it was collected during a dust outbreak, as discussed in the previous Section. Most pathogenic species were positively inter-correlated, while they were on average anti-correlated with some non-pathogenic species, as highlighted in Table 2. *Staphyloccus pettenkoferi* was significantly correlated with *S. cohnii, S. devriesei, S. hominis, Corynebacterium jeikeium*, and *Finegoldia magna*, since they may potentially cause health care-associated infections and have been detected in infections such endocarditis and sepsis (Nas et al., 2020). *S. pettenkoferi* was also significantly correlated with *Dietzia alimentaria*, a species of the genus *Dietzia* that could be a potential human pathogen in immunocompromised patients and has applications in medical, chemical, and food industries (Gharibzahedi et al., 2014). Negative significant correlations occured between *S. pettenkoferi* and the species *Gemmatirosa kalamazoonesis*, which was isolated from organically-managed agricultural soil (DeBruyn et al., 2013), and *Nitrolancea hollandica*, which is a nitrite-oxidizing bacterium from the phylum Chloroflexi, isolated from a nitrifying bioreactor (Sorokin et al., 2014). The second most abundant species *C. tuberculostearicum* showed significant positive correlations with *C. jeikeium*, *Staphylococcus devriesei*, *Acinetobacter johnsonii*, *Dietzia alimentaria*, and mainly with *Finegoldia magna*. Note that Hurst et al. (2021) characterized the nasopharyngeal microbiomes of 274 children, adolescents, and young adults with close contact with SARS-CoV-2-infected individuals, using 16S-rRNA-gene amplicon sequencing and detected the associations between *C. tuberculostearicum* and *Finegoldia magna*, in addition to the occurrence of respiratory symptoms in SARS-CoV-2 patients. *C. tuberculostearicum* presented significant negative correlations with the non-pathogenic species *Rubellimicrobium roseum*, *Blastococcus aggregatus*, and *Gemmatirosa kalamazoonesis*.

**Table 2:** Relationships between the most abundant bacterial species in the 17 analysed samples

Bacterial species	Spearman correlation coefficients	
	Positive correlations	Negative correlations
*Staphylococcus pettenkoferi*	*Staphylococcus cohnii* (0.55*), *Finegoldia magna* (0.51*), *Corynebacterium jeikeium* (0.77**), *Staphylococcus devriesei* (0.72**), *Dietzia alimentaria* (0.55*), *Staphylococcus hominis* (0.57*)	*Gemmatirosa kalamazoonesis* (− 0.49*), *Nitrolancea hollandica* (− 0.61**)
*Corynebacterium tuberculostearicum*	*Finegoldia magna* (0.70**), *Corynebacterium jeikeium* (0.49*), *Staphylococcus devriesei* (0.57*), *Dietzia alimentaria* (0.63**)	*Rubellimicrobium roseum* (− 0.64**), *Blastococcus aggregatus* (− 0.54*), *Gemmatirosa kalamazoonesis* (− 0.60*)
*Methyloversatilis universalis*	*Uncultured Acidobacteria* (0.62**), *Propionibacterium acnes* (0.62**), *Oxalophagus oxalicus* (0.82**), *Dietzia alimentari*a (0.63**)	*Uncultured eubacterium* (− 0.49*)
*Pedobacter sp.*		
*Uncultured Acidobacteria*	*Oxalophagus oxalicus* (0.51*)	*Staphylococcus cohnii* (− 0.49*), *Acinetobacter lwoffii* (− 0.58*)
*Propionibacterium acnes*	*Corynebacterium jeikeium* (0.59*), *Staphylococcus devriesei* (0.60*), *Acinetobacter johnsonii* (0.51*), *Dietzia alimentaria* (0.82**)	*Rubellimicrobium roseum* (− 0.55*), *Uncultured eubacterium* (− 0.83*), *Gemmatirosa kalamazoonesis* (− 0.49*), *Nitrolancea hollandica* (− 0.62**), *Other* (− 0.53*), *Unclassified* (− 0.54*)
*Rubellimicrobium roseum*	*Uncultured eubacterium* (0.66**), *Blastococcus aggregatus* (0.86**), *Gemmatirosa kalamazoonesis* (0.65**), *Nitrolancea hollandica* (0.59*), *Other* (0.70**)	*Finegoldia magna* (− 0.49*), *Acinetobacter lwoffii* (− 0.50*), *Corynebacterium jeikeium* (− 0.52*), *Acinetobacter johnsonii* (− 0.54*), *Dietzia alimentaria* (− 0.61**)
*Staphylococcus cohnii*	*Finegoldia magna* (0.74**), *Acinetobacter lwoffii* (0.75**),*Corynebacterium jeikeium* (0.58*), *Staphylococcus hominis* (0.60*)	*Blastococcus aggregatus* (− 0.55*), *Gemmatirosa kalamazoonesis* (− 0.72**), *Nitrolancea hollandica* (− **0.59**)
*Finegoldia magna*	*Acinetobacter lwoffii* (0.52*), *Corynebacterium jeikeium* (0.83**), *Staphylococcus devriesei* (0.52*), *Dietzia alimentaria* (0.60*)	*Blastococcus aggregatus* (− 0.59*), *Gemmatirosa kalamazoonesis* (− 0.84**), *Nitrolancea hollandica* (− 0.49*)
*Acinetobacter lwoffii*	*Corynebacterium jeikeium* (0.58*), *Staphylococcus hominis* (0.59*)	*Uncultured eubacterium* (− 0.59*), *Blastococcus aggregatus* (− 0.49*), *Others* (− 0.58*)

The related Spearman correlation coefficient is reported in brackets with values significant at a *p*-level < 0.05 and 0.01 marked by * and **, respectively. Negative correlation coefficients are in bold

The *Uncultured Acidobacteria* species showed significant positive correlations with *Methyloversatilis universalis* and *Oxalophagus oxalicus*, which is an environmental bacterial species (Rainey, 2015) and was detected in all samples of this study and at greater within-sample RAs% in HIRP_A_ (1.09%) and HIR1 (2.97%). Table 2 also shows that *Uncultured Acidobacteria* was significantly anti-correlated with the pathogenic species *Staphylococcus cohnii* and *Acinetobacter lwoffii*. In fact, *Acinetobacter spp.* are normally non-pathogenic for healthy individuals. However, in addition to *A. baumannii* and *A. johnsonii*, *A. lwoffi* can play a relevant role in nosocomial infections for debilitated patients (Rathinavelu et al., 2003).

Note also that *Propionibacterium acnes* was positively correlated with some pathogenic bacterial species (*Corynebacterium jeikeium*, *Staphylococcus devriesei*, *Acinetobacter johnsonii*, and *Dietzia alimentaria*), but it was anti-correlated with *Rubellimicrobium roseum*, *Uncultured eubacterium*, *Gemmatirosa kalamazoonesis*, *Nitrolancea hollandica*, in addition to *Others* and *Unclassified* species.

#### Analysis of richness, biodiversity and similarity among samples

The number of OTUs (Operational Taxonomic Units), the Shannon and Simpson indices (*H* and *D*, respectively), and the Principal Coordinate Analysis (PCoA) results are discussed in this Sub-section to further contribute to the characterization of the samples’ airborne bacterial community at the species level. Table 3 shows *H* and *D* values, in addition to the OTU number, for each sample. The OTU number, which is representative of the sample richness, reached the smallest value (231) in the MSR sample and varied within the 1,698–4,735 range in all the other 16 samples because of their higher richness than sample MSR. The higher value of the OTU number in the outdoor sample RB2 (4,214) than in RB1 (2,305) supports the hypothesis that RB2 was collected during a desert dust outbreak. In fact, besides increasing the crustal contribution in the atmospheric particle concentration, dust events also increase the biodiversity of the airborne bacterial community, as shown by Romano et al. (2019).

**Table 3:** Number of operational taxonomic units (n° OTUs), Shannon (*H*) and Simpson (*D*) index values at the species level for the 17 analysed samples. The sampling date of each sample is also reported

Sample	Sampling date (dd/mm/yy)	n° OTUs	At species level	
			Shannon index (*H*)	Simpson index (*D*)
HIRP_A_	30/04/2020	2104	3.94	0.12
RP_A_	01/05/2020	2737	4.21	0.13
RP1_B_	05/05/2020	2460	3.89	0.11
RP2_B_	06/05/2020	3081	4.14	0.11
BTP_B_	05/05/2020	2501	3.88	0.12
RP1_B+C_	17/05/2020	4104	4.91	0.11
RP2_B+C_	21/05/2020	4735	4.40	0.20
EF1	07/05/2020	2703	4.34	0.12
EF2	07/05/2020	2984	4.57	0.14
HIR1	01/05/2020	1698	3.83	0.11
HIR2	14/05/2020	4212	3.90	0.17
R1	02/05/2020	4334	4.28	0.22
R2	04/06/2020	3769	4.67	0.15
MSR	03/05/2020	231	2.54	0.36
RB1	08/05/2020	2305	4.45	0.14
RB2	16/07/2020	4214	4.70	0.15
PSD	11/07/2020	4569	4.48	0.17

The Shannon index *H*, which increases with the samples’ richness and evenness, varied within the 3.83–4.91 range, except in sample MSR, where it reached the smallest value equal to 2.54. The Simpson index *D*, the most common dominance measure, reached the highest value (0.36) in sample MSR, because it was the sample characterized by the smallest richness. In contrast, *D* varied within the 0.11–0.22 range in all the other samples because of the high diversity at the species level. However, both *H* and *D* index values showed a moderate variability among the 16 analysed samples, except for sample MSR.

**Fig. 4:**
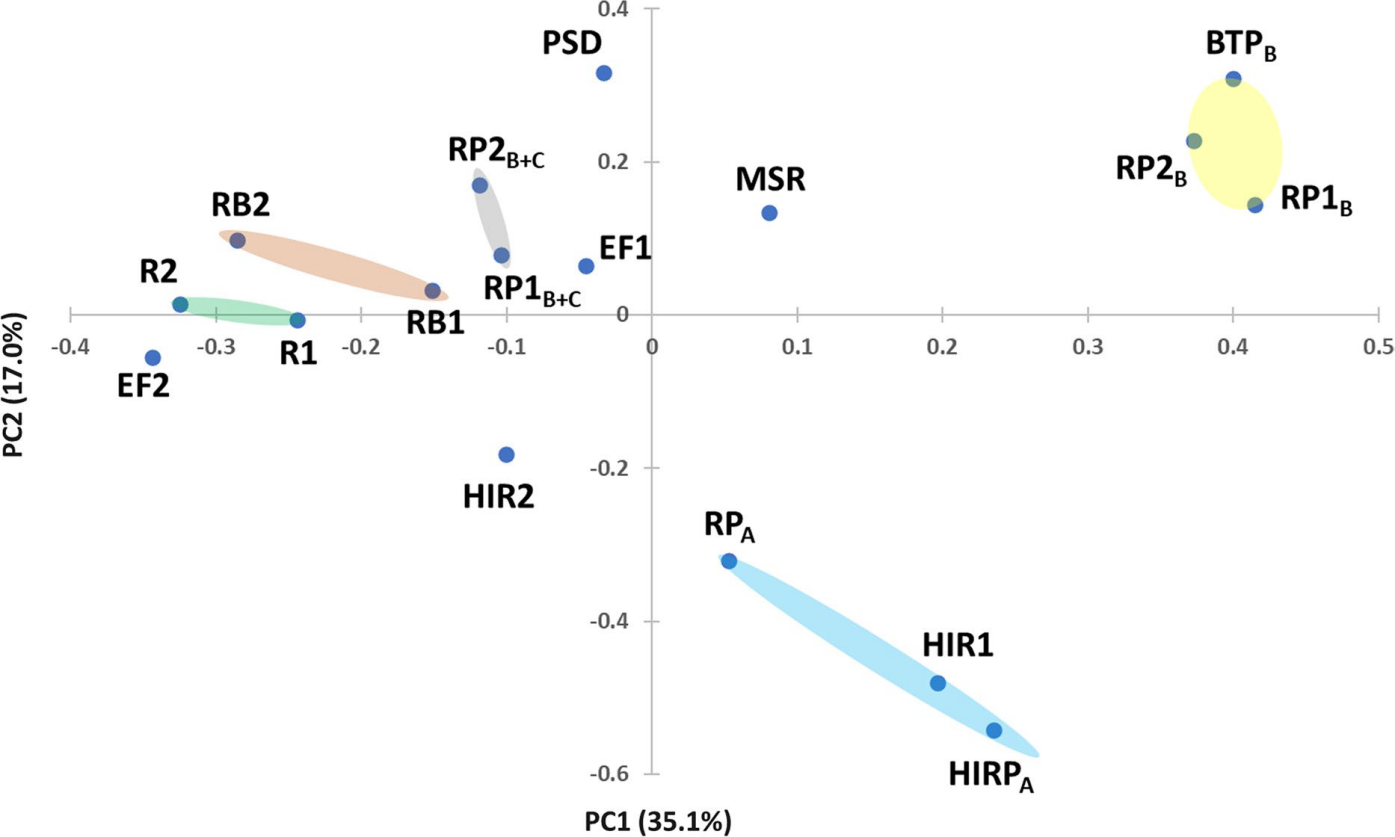
The two-dimensional Principal Coordinate Analysis (PCoA) plot based on the Bray–Curtis distance matrix for the 21 most abundant bacterial species identified in the 17 investigated samples: high isolation room with patient A (HIRP_A_), room with patient A (RP_A_), rooms with patient B (RP1_B_ and RP2_B_), bathroom of patient B (BTP_B_), rooms with patients B and C (RP1_B+C_ and RP2_B+C_), electret filters with gravimetric deposition (EF1 and EF2), high isolation rooms (HIR1 and HIR2), rooms without patients (R1 and R2), medicine store room (MSR), outdoor samplings (RB1 and RB2), and room of the Psychiatry Department (PSD). Five distinct clusters have been identified by different colours and 70% confidence ellipses. The percentages of the total variance explained by the first and second principal components are also reported in the plot

The PCoA plot based on Bray–Curtis distances of the 21 most abundant species has been calculated to further explore the similarities between the species-level bacterial structure of the analysed samples (Fig. 4). The total variance percentages explained by the first (PC1) and second (PC2) synthetic PCoA axis, which are equal to 35.1% and 17.0%, respectively, highlight a good performance of the used technique.

Since the distance between samples decreases with the similarity increase among samples in PCoA plots, Fig. 4 shows that most of the samples could be clustered into five distinct groups, each of them associated with a specific sampling site and/or environment sampling type. Different colours have been used in Fig. 4 to identify the 70% confidence ellipses associated with the five identified clusters, which are located in different areas of the PCoA plot. Samples characterized by a species-level bacterial profile significantly different from the others are spread in the PCoA graphical area. Samples RP1_B_, RP2_B_, and BTP_B_ grouped in the same confidence ellipse (reported in yellow) were all collected in the patient B areas of the ICU ward and the discussion in Sub-Sect. 3.5.1 has shown that the prevailing species are common in all these samples. In contrast, observe that sample EF2, which was also collected in the patient B room, is in a distinct region of the PCoA plot. The different type of sampling (gravimetric collection of aerosol and bioaerosol particles over 14 days for EF2) likely determined a species-level profile significantly different from the ones of the yellow cluster samples, as also shown in Fig. 3a. Sample MSR is away from all the other samples since it was collected in the less contaminated Medicine-Store Room, as previously mentioned. Samples PSD and EF1, which were collected in the Psychiatry Department and in the corridor of the ICU ward, respectively, also are away from most of the other samples because of the lower similarity in species-level profiles with the ones of the other samples, as Fig. 3a clearly shows. The light blue ellipse encloses the samples collected in the rooms occupied by patient A, in addition to sample HIR1, which was collected in the HIR without any patient. The sample HIR2 location in the PCoA plot is away from all the other samples as its bacterial species profile suggests (Fig. 2a). This last result is likely related to the 25 air changes/hour in HIR2, which likely favoured the significant dispersion/collection of the *Pedobacter sp.*, as mentioned. The samples collected in the ICU room with patients B and C are also enclosed in the same confidence ellipse (reported in grey in Fig. 4). Finally, the light brown and light green confidence ellipses enclose the outdoor-collected samples RB1 and RB2 and the samples R1 and R2 collected in ICU rooms without patients, respectively. Their close location in the PCoA plot likely shows that the species-level profile similarity of the outdoor air samples is consistent with the one of hospital rooms without any patient.

## Summary and conclusions

Indoor air samples have been collected at the ICU ward of the Operational Unit of Infectious Diseases in Galatina (Lecce, Italy), designated for the treatment of COVID-19 patients from January to June 22, 2020. Investigating the SARS-CoV-2 virus detection in air samples and contributing to the characterization and distribution of the environmental microbiome in ICU ward areas represented the main goals of this study.

- None of the air samples collected at the ICU ward of this study was found positive to SARS-CoV-2, according to the Allplex 2019-nCoV Assay analytical sensitivity (3 viral copies per microliter). This result is in reasonable accordance with some of the previous studies based on different methodologies. In fact, the airborne transmission of SARS-CoV-2 is still a subject that needs to be investigated. Presumably, rather high concentrations of aerosol particles are required to detect the airborne transmission of SARS-CoV-2 and/or the positivity of some molecular tests is insufficient to draw significant conclusions.
- Fourteen samples were collected in ICU rooms with and without COVID-19 patients. One sample was collected in the Psychiatry Department (sample PSD), and two samples (RB1 and RB2) were collected on the hospital roof for comparison.
- The 16S rRNA gene metabarcoding approach was used to investigate the samples’ environmental microbiome showing that Proteobacteria, Actinobacteria, Firmicutes, Bacteroidetes, and Acidobacteria were on average the most abundant phyla in indoor and outdoor samples, as commonly observed worldwide.
- *Staphylococcus*, *Corynebacterium*, *Sphingomonas*, and *Pseudomonas* were the most abundant genera and their within-sample RAs% varied on average significantly from sample to sample.
- The bacterial species profile allowed highlighting the main fingerprints of each single sample and/or group of samples. *Staphylococcus pettenkoferi* reached the highest within-sample RAs% (spanning the 1.54–17.31% range) in ICU patients’ rooms, suggesting that it was likely the main pathogenic species of the COVID-19 patients at the ICU ward of this study. *S. pettenkoferi* was also detected in all the others indoor and outdoor samples at within-sample RAs% < 1%. *S. cohnii*, *S. devriesei*, and *S. hominis* were also detected among the 21 most abundant species. They were dominant in RP1_B_, RP2_B_, and BTP_B_ samples, while *S. pettenkoferi* was prevailing in the HIRP_A_ (17.31%) and RP_A_ (9.30%) samples. The *CoNS* species are worldwide among the most commonly isolated bacterial species in clinical microbiology laboratories, undoubtedly mostly as skin contaminants.
- *Corynebacterium tuberculostearicum*, a ubiquitous bacterium colonizing human skin, was dominant in RP1_B_ (4.80%) RP2_B_ (4.81%), and BTP_B_ (5.73%) samples, in addition to *S. pettenkoferi*.
- The OTU and species numbers reached higher values in the RP1_B+C_ and RP2_B+C_ samples, respectively, than in the samples collected in other patient rooms. *S. pettenkoferi*, *C. tuberculostearicum*, *C. jeikeium*, and *S. hominis* were the prevailing pathogenic species in RP1_B+C_ and RP2_B+C_ samples, all existing numerously and widely in hospital environments. *Uncultured Acidobacteria*, *Rubellimicrobium roseum*, and *Uncultured eubacterium*, which contribute to the soil microbial community fraction, also reached high within-sample RAs% in both samples.
- The bacterial species profile of the samples collected in high ventilation rooms has highlighted the significant prevalence of a single bacterial species. *S. pettenkoferi* reached the highest within-sample RA% (17.31%) in the HIRP_A_ sample. Conversely, the non-pathogenic *Methyloversatilis universalis* and *Pedobacter sp.* species reached the highest within-sample RAs% in HIR1 (16.60%) and HIR2 (22.20%) sample, respectively. These last results may likely allow speculating that frequent air changes, which favour the air dispersion, may also favour the collection of prevailing airborne species during sampling.
- The correlation analysis among the 21 most abundant species has revealed that most pathogenic species were positively inter-correlated, while they were on average negatively correlated with non-pathogenic species.
- Finally, the PCoA plot based on Bray–Curtis distances of the 21 most abundant species has provided a clear view of the sample clustering according to the similarity among samples. We found that some of the samples could be clustered into distinct groups because of the high similarity among the species profile, while others were away from other samples or clusters, since each sample was associated with a specific environmental sampling condition.

In conclusion, we believe that the results of this study, which are consistent with previously published data, have contributed to the characterization of nosocomial bacterial pathogens in ICU hospitalization rooms with and without COVID-19 patients. More specifically, they have further shown that the prevailing pathogenic species in COVID-19 ICU wards were mostly skin contaminants and that their within-sample RAs% varied from a sampling site to another. The likely impact of the outdoor bacterial species profile has also been highlighted. In fact, the transport of outdoor airborne bacteria can be responsible for the spreading of pathogenic and non-pathogenic microorganisms, making the exchange of species among ecosystems possible. We are aware that the small number of the analysed samples, which prevented us from carrying out a robust statistical analysis, may represent a weakness of the paper, but bureaucratic and technical reasons did not allow us to perform additional samplings.

## Supplementary Information

Below is the link to the electronic supplementary material.

Supplementary file1 (XLSX 884 KB)

## Data Availability

Data are contained within the article or Supplementary Material.
